# CRISPR/Cas9-based genetic screen of SCNT-reprogramming resistant genes identifies critical genes for male germ cell development in mice

**DOI:** 10.1038/s41598-021-94851-9

**Published:** 2021-07-29

**Authors:** Most Sumona Akter, Masashi Hada, Daiki Shikata, Gen Watanabe, Atsuo Ogura, Shogo Matoba

**Affiliations:** 1 Bioresource Engineering Division, Bioresource Research Center, RIKEN, 3-1-1 Koyadai, Tsukuba, Ibaraki 305-0074 Japan; 2 Cooperative Division of Veterinary Sciences, Tokyo University of Agriculture and Technology, Fuchu, Tokyo 183-8509 Japan; 3 Research Fellow of Japan Society for the Promotion of Science, Tokyo, Japan; 4 Graduate School of Life and Environmental Sciences, University of Tsukuba, Tsukuba, Ibaraki 305-8572 Japan; 5 The Center for Disease Biology and Integrative Medicine, Faculty of Medicine, University of Tokyo, Tokyo, 113-0033 Japan; 6 RIKEN Cluster for Pioneering Research, Wako, Saitama 351-0198 Japan; 7 Present Address: Laboratory of Pathology and Development, Institute for Quantitative Biosciences, The University of Tokyo, Tokyo, 113-0032 Japan

**Keywords:** Germline development, Animal breeding, Development

## Abstract

Male germ cells undergo complex developmental processes eventually producing spermatozoa through spermatogenesis, although the molecular mechanisms remain largely elusive. We have previously identified somatic cell nuclear transfer-reprogramming resistant genes (SRRGs) that are highly enriched for genes essential for spermatogenesis, although many of them remain uncharacterized in knockout (KO) mice. Here, we performed a CRISPR-based genetic screen using C57BL/6N mice for five uncharacterized SRRGs (*Cox8c*, *Cox7b2*, *Tuba3a/3b*, *Faiml*, and *Gm773*), together with meiosis essential gene *Majin* as a control. RT-qPCR analysis of mouse adult tissues revealed that the five selected SRRGs were exclusively expressed in testis. Analysis of single-cell RNA-seq datasets of adult testis revealed stage-specific expression (pre-, mid-, or post-meiotic expression) in testicular germ cells. Examination of testis morphology, histology, and sperm functions in CRISPR-injected KO adult males revealed that *Cox7b2*, *Gm773*, and *Tuba3a/3b* are required for the production of normal spermatozoa. Specifically, *Cox7b2* KO mice produced poorly motile infertile spermatozoa, *Gm773* KO mice produced motile spermatozoa with limited zona penetration abilities, and *Tuba3a/3b* KO mice completely lost germ cells at the early postnatal stages. Our genetic screen focusing on SRRGs efficiently identified critical genes for male germ cell development in mice, which also provides insights into human reproductive medicine.

## Introduction

Mammalian male germ cells undergo unique and complex differentiation processes involving sex determination, epigenetic reprogramming, cell migration, and spermatogenesis finally generating motile spermatozoa^1–3^. Spermatozoa, or haploid spermatids, are solely responsible for the transmission of male genetic information to the next generation via fertilization with oocytes. In humans, it has been estimated that infertility affects 8–12% of couples globally and male factors play a primary or contributing cause in 50% of these couples^4^. Although a variety of factors could cause infertility in men, the primary cause resides in germ cell development or differentiation processes that lead to the absence of spermatozoa or abnormal spermatozoa^4,5^. Although assisted reproductive techniques such as intracytoplasmic sperm injection or round spermatid injection could partly overcome these problems^6,7^, it is important to understand the mechanisms of infertility to develop new technologies to support reproduction. Despite such importance, the mechanisms of these dynamic processes of male germ cell development remain elusive.

A previous in silico survey of gene expression datasets estimated that more than 2,300 genes were predominantly expressed in male germ cells^8^. Indeed, dozens of genes have been identified that play essential roles in male germ cell development or fertility mainly using mouse genetic models. Recently, the genome-editing technology, CRISPR/Cas9^9–11^, has enhanced the speed of genetic screening^12,13^. For example, the groups of Ikawa and Matzuk have been performing extensive mouse genetic screening using the CRISPR/Cas9 system and have identified many genes that are essential for male fertility or spermatogenesis^14–17^. However, they and others also found that the great majority of testis-expressed genes are individually dispensable for spermatogenesis or male fertility^14,18–22^, which hampers the efficient identification of physiologically critical genes. Therefore, it is ideal to have a unique strategy that efficiently narrows down functionally important candidates before performing the real genetic screen.

**Figure 1:**
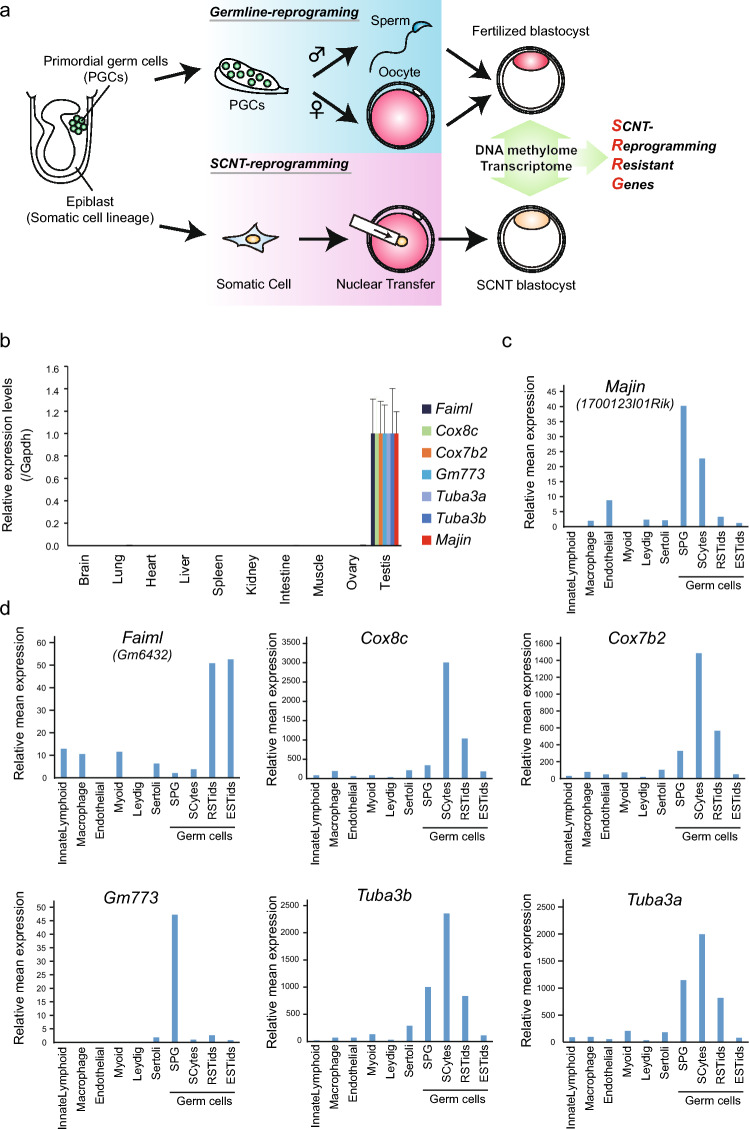
Testicular germ cell-specific expression of target SRRGs. (**a**) Schematic illustration comparing germline reprogramming and somatic cell nuclear transfer (SCNT) reprogramming and the strategy to identify SCNT-reprogramming resistant genes (SRRGs). Germ cells that are differentiated from epiblast as primordial germ cells undergo germline-specific epigenetic reprogramming through either spermatogenesis or oogenesis. In SCNT, on the other hand, the somatic cell genome skips germline reprogramming, instead undergoing SCNT reprogramming in the ooplasm. Therefore, it is assumed that SCNT embryos possess epigenetic abnormalities that are resistant to SCNT reprogramming, but are successfully reprogrammed only in the germline (see Supplementary Table S1 and Matoba et al.^25^). (**b**) RT-qPCR analysis of gene expression levels of target SRRGs and *Majin* in adult mouse tissues. Data shown are mean expression values relative to *Gapdh*. The expression level of each gene was normalized for testis as 1. (**c**,**d**) Bar graphs showing the gene expression levels of *Majin* (**c**) and target SRRGs (**d**) in multiple cell types of adult testes. Single-cell RNA-seq data were obtained from Green et al.^32^. SPG, spermatogonia; SCytes, spermatocytes; RSTids, round spermatids; ESTids, elongated spermatids. Note that *Majin* and SRRGs are highly expressed in germ cells in a stage-specific manner.

Germ cells separate from somatic cell lineages at the early stage of embryonic development^23^. Thus, when the somatic cell genome is transferred to enucleated oocytes by somatic cell nuclear transfer (SCNT), the reconstructed genome skips the epigenetic reprogramming steps that normally occur during germ cell development^24^. We have previously performed a comprehensive comparison of transcriptome and epigenome between normally fertilized blastocysts and SCNT-generated blastocysts to identify SCNT-reprogramming resistant genes (SRRGs; Fig. 1a)^25^. Interestingly, this list of SRRGs was highly enriched with the genes known to be essential for spermatogenesis in mouse KO models such as *Asz1*^26^, *Tex12*^27^, *Slc25a31*^28^, *Tex101*^15,29^, *Mael*^30^, or *Majin*^31^. For example, *Asz1* KO males are sterile due to a block in spermatid development^26^, *Tex12* KO males exhibit infertility associated with failure of crossover events during meiosis^27^, and *Majin* KO males show infertility because of the failure of meiotic telomere tethering^31^ (see Supplementary Table S1 online). Nonetheless, about half of the SRRGs were not characterized by the KO mouse model. Although these included multiple X-linked genes with a high copy number of family genes (*Mage* and *Xlr* family) that are difficult to disrupt using CRISPR/Cas9 simultaneously, some were unique genes, which indicates that the genetic screen by CRISPR/Cas9 targeting is achievable.

In this study, we have selected five candidates from uncharacterized SRRGs and performed a genetic screen using a CRISPR/Cas9-based approach to dissect their functions in male germ cell development or spermatogenesis as well as fertilization. We find that among the five candidates, three were indeed critical for male germ cell development or spermatogenesis in a time-dependent manner. Our study provides not only a new list of genes that are required for male germ cell development, but also the concept that an SCNT-reprogramming-resistant feature can be used as an efficient selection method to identify the list of germline essential genes.

## Results

### Testicular germ cell-specific expression of SRRGs

Twenty-nine SRRGs were identified by the comparison of DNA methylome and transcriptome between fertilized blastocysts and SCNT blastocysts (DNA hypermethylated at the promoter [absolute methylation level > 5%] and transcriptionally repressed in SCNT blastocysts [Fold change > 2]; see Fig. 1a and Supplementary Table S1 online). Among them, we selected five SRRGs that are not characterized in knockout (KO) mouse studies—*Faiml (Gm6432)*, *Cox8c*, *Cox7b2*, *Tuba3b*, and *Gm773*—as our screening targets. As a positive control, we also included *Majin*, one of the well-characterized SRRGs required for meiosis in mice^31^, for all the following gene expression analyses as well as screening procedures. First, we analyzed the expression patterns of the six selected genes in different adult mouse tissues using RT-qPCR. We found that all these genes were predominantly expressed in testis (Fig. 1b). To understand the cell type-specific expression patterns of these genes in the testis, we analyzed single-cell RNA-seq datasets of adult mouse testis published by Green et al.^32^. We found that all six genes are highly expressed in germ cells, but are very low in somatic cells of the adult testis (Fig. 1c,d). Moreover, while *Majin* and *Gm773* are highly expressed in premeiotic spermatogonia, *Cox8c*, *Cox7b2*, and *Tuba3b* showed the highest expression in spermatocytes. *Faiml* showed the highest expression in post-meiotic round or elongated spermatids. These results indicate that the six SRRGs, including *Majin*, are highly expressed in testicular germ cells, and suggest that SRRGs may play critical roles in mouse spermatogenesis.

### Generation of SRRG KO founder mice using CRISPR

**Figure 2:**
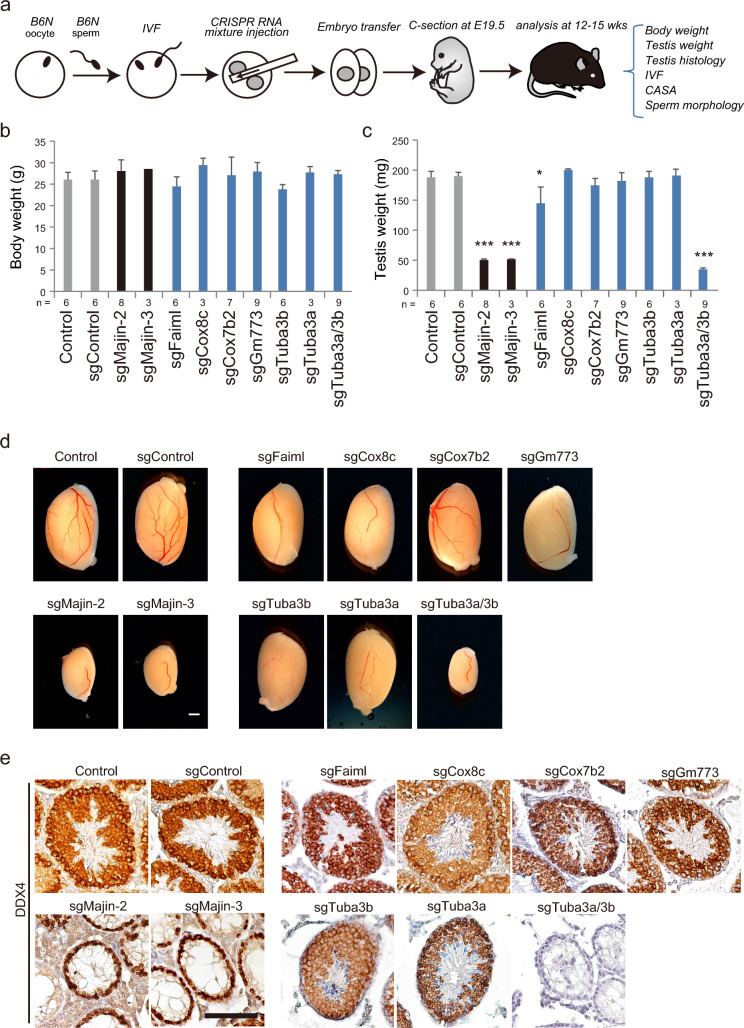
CRISPR-based genetic screening of five SRRGs. (**a**) Schematic illustration of the CRISPR-based screening procedures. C57BL/6N (B6N) oocytes and sperm were in vitro fertilized to generate B6N inbred zygotes. CRISPR mixture (*Cas9* mRNA + sgRNAs) was injected into the cytoplasm of the zygotes at 6 h post insemination (hpi). Embryos developed to the two-cell stage were transferred to the oviduct of pseudopregnant females. The embryos were recovered at E19.5 by caesarian section (C-section). The phenotypes of founder mice were screened for the indicated parameters at 12–15 weeks of age. CASA, computer-assisted sperm analysis. (**b**,**c**) Bar graphs showing the body weight (**b**) and testis weight (**c**) of adult founder mice. The testis weight was greatly reduced in sgMajin-2, sgMajin-3, and sgTuba3a/3b mice, while it was slightly reduced in sgFaiml mice. *P < 0.05, ***P < 0.001. (**d**) Gross morphology of founder testes. Note that sgMajin-2, sgMajin-3, and sgTuba3a/3b mice had significantly smaller testes than control. Scale bar, 1 mm. (**e**) Representative immunostaining images of testis sections stained against a germ cell-specific marker, DDX4. Note that while germ cells are depleted after meiotic stages in sgMajin-2 and sgMajin-3 testis, they are completely absent in sgTuba3a/3b testis. Scale bar, 100 μm.

**Figure 3:**
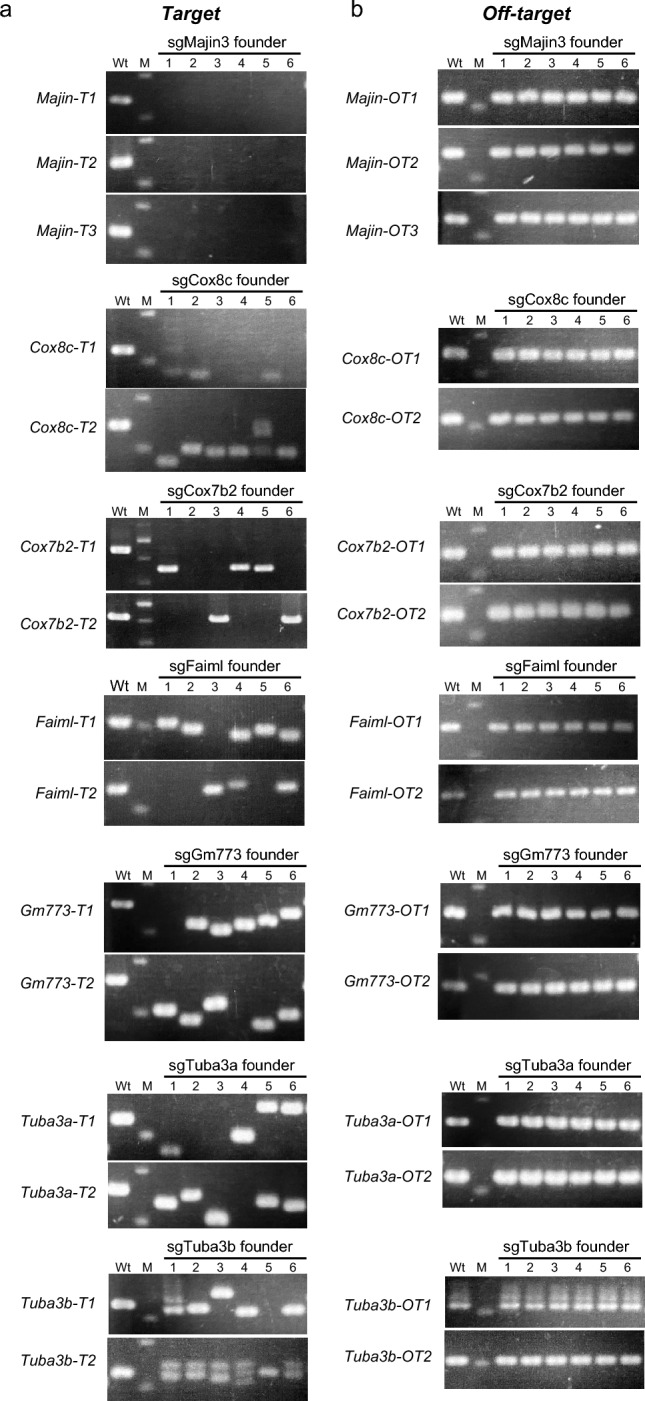
Highly efficient induction of indels at the target sites of the founder mice. (**a**) Electrophoresis images of genotyping PCR using pairs of primers that surround target sites of each sgRNA. Note that essentially all founders analyzed appeared to have biallelic indels at the target SRRGs. (**b**) Electrophoresis images of genotyping PCR using pairs of primers that surround the top ranked putative off-target site of each sgRNA. No apparent mutation was found at these off-target sites. M, marker (100 bp ladder); Wt, wild type. Full-length gels are presented in Supplementary Figure S1.

To determine the biological roles of SRRGs, we generated KO mice of each SRRG using the Triple-CRISPR-based method^13,33^. This method efficiently disrupts the function of target genes biallelically by targeting multiple sites of protein-coding exons and enables direct analysis of founder mice. Thus, we injected *Cas9* mRNA together with multiple sgRNAs targeting protein-coding exons of SRRGs into the zygotes of the C57BL/6N (B6N) strain. The CRISPR-injected embryos were transferred to pseudopregnant females and delivered at E19.5 by caesarian section (Fig. 2a). Genotyping PCR confirmed that insert/deletions (indels) were highly efficiently induced at the target sites of sgRNAs in each founder, while no apparent indel was observed at the off-target sites (Fig. 3a,b, Supplementary Fig. S1a,b online). Founder mice, grown to adult (12–15 weeks), were subjected to the spermatogenesis screening pipeline for the following parameters: body weight, testis weight, testis histology, in vitro fertilization (IVF), computer-assisted sperm analysis (CASA), and sperm morphology (Fig. 2a). To speed up the screening, we did not apply a natural mating-based fertility test, which involves up to several months of mating with females. Instead, we performed IVF to evaluate quickly the fertility of spermatozoa. First, we questioned whether two or three sgRNAs could disrupt the function of target genes by targeting *Majin*. *Majin* KO mice have been shown to display meiotic arrest during spermatogenesis, but no other phenotype including aberrations of embryonic development or postnatal growth, in male mice^31^. Consistent with that KO report^31^, embryos injected with three sgRNAs against *Majin* (sgMajin-3) showed no growth defects in body size (Fig. 2b), but a severe reduction in testis size (Fig. 2c,d). Importantly, founders generated by two sgRNAs against *Majin* (sgMajin-2) also showed a similar phenotype with sgMajin-3 founders at a consistent efficiency (Fig. 2b–d). We next asked if the germ cells are present in these *Majin* KO mice by immunostaining against mouse VASA homolog (DDX4), which is widely expressed in the testicular germ cells^34^. We then found that DDX4 positive germ cells were present but arrested during meiosis in all sgMajin-2 and sgMajin-3 mice analyzed (Fig. 2e). These results indicate that two sgRNAs could efficiently induce a KO phenotype similar to three sgRNAs. Because it was difficult to design three sgRNAs with high specificity for some target SRRGs, we applied the two sgRNAs approach to the screening of other SRRGs.

### Spermatozoa of sgCox7b2 mice failed to fertilize under IVF due to their poor motility

**Figure 4:**
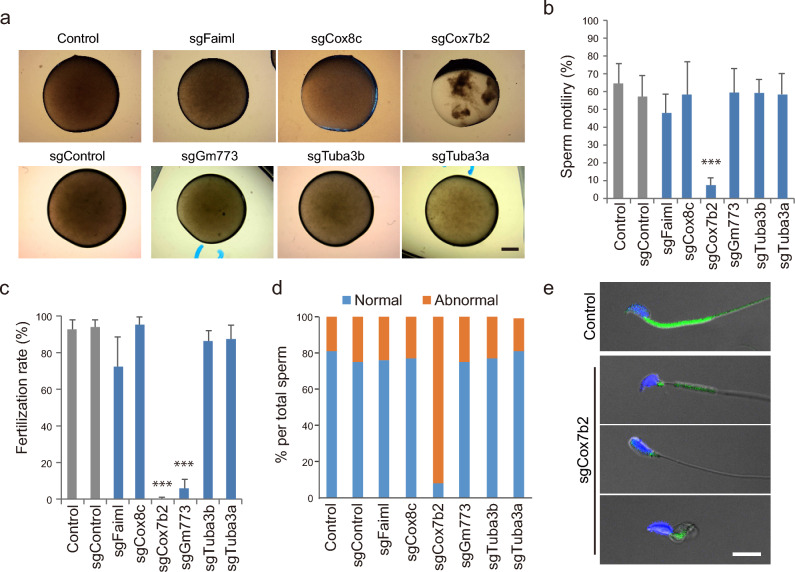
Sperm fertility, motility, and morphology analyses of SRRG KO mice. (**a**) Representative images of HTF drops suspended with epididymis-isolated spermatozoa at 1 h of culture. Scale bar, 1 mm. Note that sperm clots of sgCox7b2 failed to spread throughout the drop and remained aggregated. (**b**) Bar graphs showing the sperm motility examined by CASA at 1 h after preincubation. The motility was severely reduced in sgCox7b2 spermatozoa. *** *P* < 0.001. See Supplementary Videos S1 and S2 online. (**c**) Bar graphs showing the fertilization rate of spermatozoa of founder males with B6N oocytes by IVF. Fertilized zygotes were identified by the presence of two pronuclei at 5–6 h after insemination. ***P < 0.001. (**d**) Bar graphs showing the ratio of sperm with normal or abnormal morphology. Note that the great majority of spermatozoa showed abnormal morphology in sgCox7b2 testis. (**e**) Representative images of spermatozoa of control and sgCox7b2 mice stained with Hoechst 33342 (DNA; blue) and Rhodamine 123 (Mitochondria; green). sgCox7b2 spermatozoa displayed abnormality in the head and midpiece. The signals of Rhodamine 123 were severely reduced and disorganized in sgCox7b2 spermatozoa. Scale bar, 10 μm.

Knockout mice of the five target SRRGs developed normally and grew to adults (Supplementary Table S2 online). Consistent with their testis-specific expression (Fig. 1b), these SRRG KO female mice showed normal fertility by IVF and normally produced pups after embryo transfer (Supplementary Table S3 online). By contrast, sgMajin-2 and sgMajin-3 females did not ovulate due to the absence of oocytes, as reported^31^, validating the reliability of our approach (Supplementary Table S3 online). During measurements of testis size or histology analyses, we did not detect any significant defects in the five SRRG KO males (Fig. 2d,e). Therefore, we next examined the ability of spermatozoa to fertilize oocytes. The sperm clot was isolated from the epididymis and suspended in a drop of culture medium (human tubal fluid; HTF medium) to allow sperm to swim up and capacitate. After 1 h of incubation, the control sperm actively swam to distribute evenly within the drop (Fig. 4a, Supplementary Video S1 online). Because spermatogenesis was completely arrested at meiotic stages in sgMajin-2 and sgMajin-3 mice (Fig. 2e), we could not obtain spermatozoa from the epididymis of these mice. Spermatozoa isolated from other SRRG KO mice actively swam up and evenly distributed in the drop, except for those of sgCox7b2 mice. The spermatozoa of sgCox7b2 mice remained aggregated in the HTF drop even after 1 h of incubation and showed very poor motility (Fig. 4a, Supplementary Video S2 online). Indeed, CASA analysis revealed that sperm motility was severely reduced in sgCox7b2 sperm (Fig. 4b). Consistent with such severely reduced motility, spermatozoa of sgCox7b2 mice failed to fertilize with oocytes under IVF (Fig. 4c). We further analyzed the morphology of spermatozoa by DNA/mitochondria (Hoechst 33342/Rhodamine 123) staining and found that most sperm of sgCox7b2 mice displayed abnormal morphology in their head (58%) and/or midpiece (92%) (Fig. 4d,e). In particular, mitochondria that normally aligned at the midpiece appeared to be severely disorganized or reduced in these sgCox7b2 spermatozoa (Fig. 4e). These results suggest that *Cox7b2* plays essential roles in spermatogenesis, especially at the last step of spermiogenesis to mature into functional spermatozoa.

### sgGm773 spermatozoa showed reduced fertility due to the poor capability to penetrate the zona pellucida

**Figure 5:**
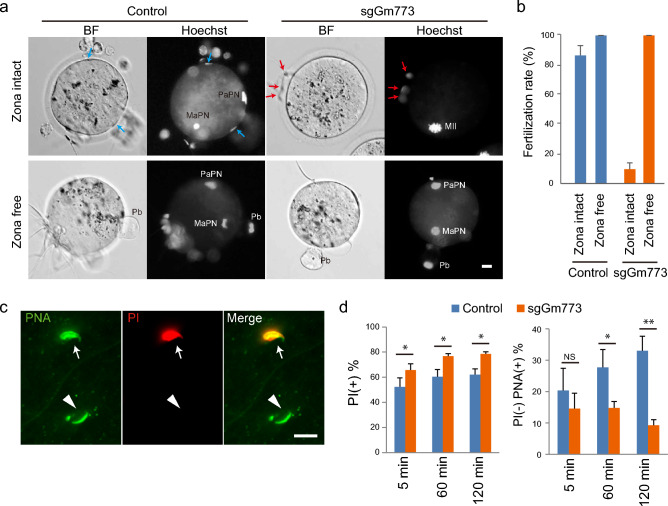
Defects of sgGm773 spermatozoa related to their poor fertility. (**a**) Representative images of oocytes and spermatozoa at 1 h after insemination by IVF. Zona-free oocytes were prepared by brief treatment with acid Tyrode. DNA was stained with Hoechst 33258. Blue arrows, sperm heads penetrated the perivitelline space. Red arrows, spermatozoa stacked on the surface of zona pellucida. BF, bright field; PaPN, paternal pronucleus; MaPN, maternal pronucleus; MII, MII stage spindle; Pb, polar body; Scale bar, 10 μm. (**b**) Bar graphs showing the fertilization rate of control and sgGm773 spermatozoa inseminated with zona intact or zona-free oocytes. The fertilization rate remained at less than 10% when sgGm773 spermatozoa were inseminated with zona intact oocytes and increased to 100% when zona pellucida was removed. (**c**) Representative images of sperm head stained with PI or PNA. Arrows indicate PI(+) spermatozoon. Arrowheads indicate PI(−)/PNA(+) spermatozoon. Scale bar, 10 μm. (**d**) Bar graphs showing the percentage of PI(+) and PI(−)/PNA(+) spermatozoa at the indicated time points of preincubation.

Although spermatozoa of all SRRG KO mice, except for sgCox7b2, showed normal motility (Fig. 4b), sgGm773 spermatozoa showed significantly reduced fertility to less than 10% by IVF (Fig. 4c). Careful examination of oocytes after IVF revealed that, while many spermatozoa of control mice could penetrate through the zona pellucida (Fig. 5a; blue arrows), the great majority of sgGm773 spermatozoa failed to penetrate and stacked up at the surface of the zona pellucida (Fig. 5a; red arrows). Therefore, we next examined whether the sgGm773 spermatozoa can efficiently fertilize with oocytes when the zona pellucida is removed. After zona removal by acid Tyrode treatment, sgGm773 spermatozoa fertilized with the nude oocytes at 100% efficiency (Fig. 5b). These results suggest that sgGm773 spermatozoa might have a problem in zona pellucida-penetration activity, although their motility and morphology appeared normal (Fig. 4b,d). Therefore, we next examined the integrity of the sperm plasma membrane and acrosome by staining with propidium iodide (PI) and peanut agglutinin (PNA), respectively, as we reported previously (Fig. 5c)^35^. PI staining allows us to distinguish between membrane damaged (PI(+)) and intact (PI(−)) spermatozoa. Because PNA stains the acrosome, the PI(–)/PNA(+) sperm represents those that are alive and ready for the acrosome reaction. We observed similar levels of PI(+) and PI(−)/PNA(+) population ratio in control, as shown in the previous study^35^. We found that significantly more of the sgGm773 sperm was PI(+) compared with control sperm at all time points analyzed. Furthermore, the PI(−)/PNA(+) population ratio gradually decreased during the preincubation period of spermatozoa (Fig. 5d). These results suggest that sgGm773 spermatozoa showed severely reduced fertility, likely because of the poor integrity of their plasma membrane as well as a reduced acrosome reaction, both of which are required for efficient penetration through the zona pellucida.

### Germ cells are completely lost at the early postnatal stages in *Tuba3a/3b* double KO founder mice

**Figure 6:**
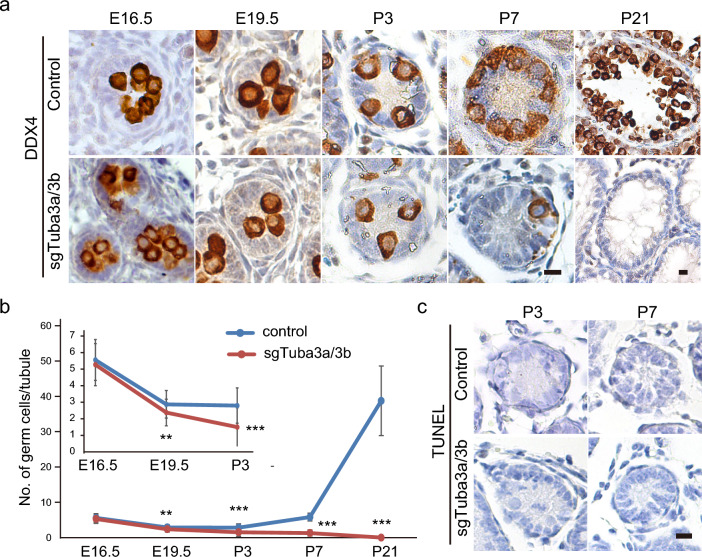
Complete depletion of gonocytes at the early postnatal stages in sgTuba3a/3b mice. (**a**) Representative images of embryonic and postnatal testis sections immunostained against DDX4 (germ cell marker). Scale bar, 10 μm. (**b**) Bar graphs showing the average number of DDX4-positive germ cells that survived in each seminiferous tubule. (**c**) Representative images of TUNEL-stained postnatal testis sections. Scale bar, 10 μm.

Although sgTuba3b mice did not show any defects on screening, we found that *Tuba3b* has a paralogue gene, *Tuba3a*, which encodes a protein TUBA3A having the same amino acid sequence as TUBA3B that is coded by *Tuba3b*. *Tuba3a* is expressed exclusively in testicular germ cells, similar to *Tuba3b* (Fig. 1b,d), suggesting that *Tuba3a* may compensate for the function of *Tuba3b* in sgTuba3b testis. Moreover, among seven Tubulin alpha (*Tuba*) genes present in the mouse genome, *Tuba3a* and *Tuba3b* are the only ones expressed in adult male germ cells (Supplementary Fig. S2a online). Thus, we generated *Tuba3a* KO mice and *Tuba3a/3b* double KO mice by injecting *Cas9* mRNA together with two sgRNAs against *Tuba3a* (sgTuba3a) or both *Tuba3a* and *Tuba3b* (sgTuba3a/3b), respectively. Both sgTuba3a and sgTuba3a/3b founder mice grew normally to adulthood and females had normal fertility (Fig. 2b, Supplementary Tables S2 and S3). The sgTuba3a male did not show any defects in all steps of screening, similar to sgTuba3b males. However, the testes of adult sgTuba3a/3b founder mice were strikingly small (Fig. 2c,d). Histological examination revealed that sgTuba3a/3b testes completely lack DDX4-positive germ cells at 12 weeks of age (Fig. 2e). We next asked at what stage do germ cells disappear in sgTuba3a/3b testes during development. The DDX4-positive gonocytes were normally present in the sgTuba3a/3b testis at E16.5 (Fig. 6a). However, the number of gonocytes was slightly reduced at the newborn stage. Thereafter, gonocytes of sgTuba3a/3b mice were gradually depleted during the postnatal growth period between P3 and P7 and completely lost by 3 weeks after birth (Fig. 6a,b). During these stages, we did not observe any TUNEL signals, which represent apoptosis in sgTuba3a/3b gonocytes (Fig. 6c). These results suggest that *Tuba3a/3b* plays a critical role in the survival of male germ cells during the early postnatal growth period.

## Discussion

**Figure 7:**
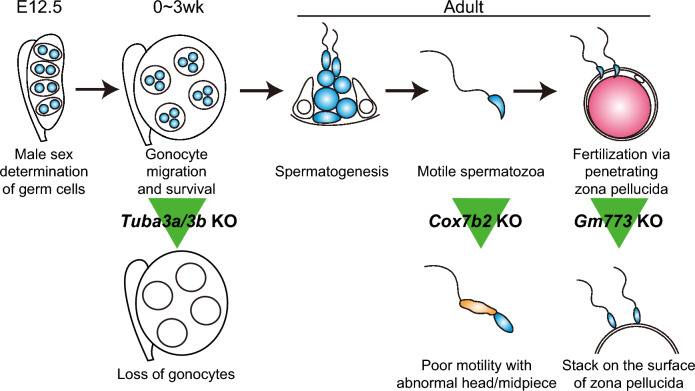
SRRG KO caused variable defects during male germ cell development. Schematic illustration of male germ cell development and the phenotypes observed in the SRRG KO mice in this study. *Tuba3a/3b* KO caused complete depletion of gonocytes at the early postnatal stages. *Cox7b2 KO* caused poor motility of spermatozoa, likely because of the abnormal morphology in the midpiece of spermatozoa. *Gm773* KO caused a significant reduction of sperm fertility, likely because of the poor penetration capacity through zona pellucida.

In this study, we focused on a unique list of SRRGs that are predominantly expressed in male germ cells and performed a CRISPR-based genetic screen to examine their physiological functions in mice. Although there are more than 2,300 genes that show predominant expression in male germ cells, this list of SRRGs appears to be enriched for physiologically important genes based on the available KO reports (germline defects are observed in 10 out of the 13 SRRG genes for which KO mice have been reported; see Fig. 1a and Supplementary Table S1). Indeed, we demonstrated that among five screened SRRGs, three were essential for the male germ cell development at different time points. Specifically, *Tuba3a* and *Tuba3b* are required for the survival of gonocytes during postnatal growth, *Cox7b2* is required for the completion of spermiogenesis to mature into motile sperm, and *Gm773* plays a critical role in the acquisition of zona pellucida-penetration capacity (Fig. 7). Our strategy of directly analyzing founder mice generated by CRISPR-targeting to the protein-coding exons can efficiently reveal the function of candidate genes^13^. In particular, this strategy is very powerful when multiple genes may complement each other because it allows these genes to be knocked out simultaneously. Using this strategy, we successfully generated a double KO for *Tuba3a* and *Tuba3b* in founder mice that showed highly consistent phenotypes among different founders. Thus, our approach to disrupt multiple genes simultaneously using CRISPR may be useful for dissecting the roles of redundant genes.

The initial list of SRRGs included two cytochrome c oxidase (COX) genes, *Cox8c* and *Cox7b2*. COX, or Complex IV, is a large transmembrane protein complex that is responsible for the terminal step in the mitochondrial electron transport chain that drives oxidative phosphorylation^36^. COX is composed of 13 subunits, three of which are encoded by mitochondrial DNA, while the other 10 are from nuclear DNA. Among these 13 subunits, COX7B2 forms subunit VIIb, which could be made from either of two nuclear genes, *Cox7b* or *Cox7b2*, in mammals. Based on the single-cell RNA-seq data of adult testis, testicular germ cells express *Cox7b2*, but not *Cox7b* (Supplementary Fig. S2d online). Similarly, among three *Cox8* family genes (*Cox8a*, *Cox8b*, and *Cox8c*), only *Cox8c* is expressed in testicular germ cells (Supplementary Fig. S2e online). Therefore, in testicular germ cells, COX subunits VIIb and VIII are likely composed of COX7b2/*Cox7b2* and COX8C/*Cox8c*, respectively. In our screening, sgCox7b2 spermatozoa were morphologically abnormal and their motility was severely reduced, which led to complete infertility by IVF, whereas sgCox8c mice did not show any defects. It is interesting that the COX VIII subunit is dispensable, while the VIIb subunit is critically important for spermatozoa. Nuclear-coded COX subunits, including *Cox8c* and *Cox7b2*, are generally thought to regulate the assembly of mitochondria-coded core subunits of COX. Thus, *Cox7b2*/COX7B2 might play a unique role in COX assembly during spermatogenesis. It is also interesting the way abnormal COX assembly affected the morphogenesis of sperm heads, as observed in sCox7b2 spermatozoa. Further detailed examination of *Cox7b2* KO sperm, focusing on mitochondrial COX assembly, may reveal the relationship between Cox genes and sperm mitochondrial organization as well as sperm head morphogenesis.

Poor zona penetration activity leading to reduced fertility was shown by sgGm773 spermatozoa. The Gm773 protein possesses a Cor1/Xlr/Xmr conserved domain that is present in meiosis-related XLR proteins^37^. Thus, *Gm773* was expected to play critical roles during meiosis. However, sgGm773 mice did not show any apparent defects in meiosis. Instead, the spermatozoa of sgGm773 showed poor zona penetration activity, likely because of defects in the integrity of the cytoplasmic membrane or acrosome. This indicates that Gm773 may play a unique role during spermatogenesis other than meiosis. Indeed, gene expression of Gm773 was not observed in meiotic spermatocytes, but rather, was specific to spermatogonia (Fig. 1b). How GM773 expressed in spermatogonia safeguards the integrity of spermatozoa membrane/acrosome would be of interest for future study.

Germ cells were completely lost in sgTuba3a/3b mice at the early postnatal stages, while sgTuba3a and sgTuba3b did not show any clear phenotype. *Tuba3a* and *Tuba3b* encode Tubulin alpha proteins. The *Tuba* gene family in mice consists of seven members: *Tuba1a*, *Tuba1b*, *Tuba1c*, *Tuba3a*, *Tuba3b*, *Tuba4a*, and *Tuba8*. Although each TUBA protein has several unique residues in its amino acid sequence, those of TUBA3A and TUBA3B are the same (they are paralogue genes). Thus, each gene appears to compensate for the function of another gene in individual KO mice. Indeed, the expression dynamics of *Tuba3a* and *Tuba3b* are synchronized, initiated from the middle embryonic stages (from E12.5 to E14.5), and become predominant among the *Tuba* family after birth (Supplementary Fig. S2b and S2c online). Since other *Tuba* genes are downregulated postnatally, it is speculated that tubulin alpha proteins are gradually reduced and eventually lost in the postnatal gonocytes of sgTuba3a/3b testis. Given that microtubules play critical roles in basic cellular processes including cell division, such loss of alpha tubulin in sgTuba3a/3b gonocytes may have induced collapse of basic cellular homeostasis leading to germ cell elimination, independent of apoptosis around P3 to P7. It would also be interesting to understand how the transcriptional transition of tubulin alpha genes from others to *Tuba3a/3b* around birth is epigenetically regulated.

In summary, we have provided genetic evidence that among the five SRRGs we screened, three (*Cox7b2*, *Gm773*, and *Tuba3a/3b*) are required for normal male germ cell development and maturation in mice. Although knockout of the other two genes (*Faiml* and *Cox8c*) did not show any clear phenotype in our screening criteria, we cannot exclude the possibility that these genes still play critical roles in male fertility because our screen did not include natural mating-based fertility tests. Indeed, several genes are reported to be required for the steps that are present in natural mating processes but circumvented in IVF procedures such as entry into the oviduct^15,29^. Also, there remains a possibility that the founder mice we generated might still have functional proteins, as we did not confirm a complete lack of functional proteins in each founder, although this possibility is very low considering the highly efficient induction of indels at target sites (Fig. 3a and Supplementary Fig. S1a). Although *Gm773* appears to be conserved only among rodents (mouse and rat), *Cox7b2* and *Tuba3a/3b* are widely conserved in mammals, including humans (NCBI Gene database). Importantly, human homologues of these genes (*COX7B2* and *TUBA3E*) show testis-specific expression patterns in humans similar to those in mice (Supplementary Fig. S2f,g online), suggesting that these genes might also play critical roles in male germ cell development in humans. Further detailed analyses of KO mice may reveal detailed molecular mechanisms underlying each defective process that would provide important insights into human reproductive medicine.

## Materials and methods

### Mice

C57BL/6 N (B6N) and ICR mice were purchased from SLC, Inc. (Shizuoka, Japan). Mice were housed under controlled lighting conditions (daily light from 07:00 to 21:00). All experimental protocols were approved by the Institutional Animal Care and Use Committee of RIKEN Tsukuba Institute and conducted in accordance with the Principles of Laboratory Animal Care. Animal care and use were conducted in compliance with the ARRIVE guidelines.

### RT-qPCR

Total RNA was purified from adult mouse tissues using RNeasy Mini Kits (#74,104; Qiagen, Tokyo, Japan). Thereafter, cDNAs were synthesized from the purified RNAs using the SuperScript IV First-Strand Synthesis System (#18091050; Thermo Fisher Scientific, Tokyo, Japan). RT-qPCR was then performed using PowerUp SYBR Green Master Mix (#A25742; Thermo Fisher Scientific) with the QuantStudio 7 system (Thermo Fisher Scientific). The Ct values were normalized to that of glyceraldehyde 3-phosphate dehydrogenase (*Gapdh*). The expression level of each gene was further normalized for testis as 1. The primers used for RT-qPCR are listed in Supplementary Table S4 online.

### Single-cell RNA-seq analysis

Read count matrix and clustering list of single-cell RNA-seq data for adult testis were obtained from GSE112393^32^. Read counts were transferred to counts per million (CPM) and the mean CPM of each cluster was computed using R (version 3.6.1)^38^.

### IVF

Adult (over 10 weeks of age) wild-type B6N or CRISPR founder mice were used for IVF. Briefly, spermatozoa were collected from the epididymis of adult males and incubated in the HTF drops for 1 h before insemination for their activation. Cumulus-oocyte complexes (COCs) were collected from the oviducts of B6N females that were superovulated by injecting 7.5 IU of pregnant mare serum gonadotropin (#367222; Merk Millipore, Tokyo, Japan) plus anti-inhibin serum and 7.5 IU of human chorionic gonadotropin (hCG; Millipore #230734). At 15–17 h after the hCG injection, the isolated COCs were incubated in HTF containing 1.25 mM reduced glutathione (GSH) for 1 h before insemination. After preincubation, the activated spermatozoa were introduced to the COC-containing HTF drops to initiate insemination. In some experiments, the zona pellucida was removed by treatment with acid Tyrode. At 5–6 h after insemination, the fertilized zygotes were washed in potassium simplex optimized medium (KSOM) drops.

### KO mouse production by the CRISPR/Cas9 system

Triple CRISPR-based KO mouse production was performed as described previously^13,33^. Briefly, two or three sgRNAs targeting distinct exons of each target gene were designed using the MIT CRISPR Designer (currently closed) or CRISPOR (http://crispor.tefor.net/). Sequences complementary to sgRNAs were cloned into the px330 vector by oligo annealing. The T7 promoter was added to sgRNA templates using PCR, and sgRNAs were synthesized via in vitro transcription from the PCR products as templates using a MEGAshortscript T7 Transcription Kit (Thermo Fisher Scientific). Some sgRNAs were synthesized using the GeneArt Precision gRNA Synthesis Kit (Thermo Fisher Scientific). The CRISPR target sequences and primers to generate sgRNAs are listed in Supplementary Table S5 online. *Cas9* mRNA was synthesized from pcDNA3-T7-NLS hCas9-pA vector (RIKEN, RDB13130) using mMESSAGE mMACHINE T7 ULTRA Transcription Kit (Thermo Fisher Scientific). Synthesized sgRNAs and *Cas9* mRNA were adjusted to 500 ng/μL and aliquots were frozen at –80 °C until use. The mixture of two or three distinct sgRNAs (50 ng/μL each) targeting coding exons and *Cas9* mRNA (100 ng/μL) were injected into zygotes using a piezo-driven micromanipulator at 5–6 h post-insemination.

### Embryo transfer

Two-cell stage embryos were transferred to the oviducts of pseudopregnant (E0.5) ICR females. The pups were recovered by caesarian section on the day of delivery (E19.5) and nursed by lactating ICR females.

### Immunohistochemistry

All specimens (neonatal and adult testes) were fixed overnight in 4% paraformaldehyde in phosphate buffered saline (PBS) solution and then embedded in paraffin wax after dehydration. Embedded samples were sectioned as 4 μm. After deparaffinization and rehydration, antigen retrieval was performed using sodium citrate buffer (pH 6.0) pretreated in a microwave for 10 min to maintain a sub-boiling temperature. Incubation in 3% hydrogen peroxide was carried out to block endogenous peroxidase. Nonspecific staining was blocked by 1% bovine serum albumin for 1 h at room temperature. Then, the sections were incubated overnight with primary antibody (rabbit anti-Ddx4 [#Ab13840; Abcam, Tokyo, Japan], 1:500 dilution) at 4 °C. Negative controls were carried out in the absence of a primary antibody. After that, the sections were washed three times in PBS with 0.05% Tween-20. The sections were then exposed to the biotin-labeled second antibodies for 1 h and then a Vectastain Elite ABC Kit (Vector Laboratories, Burlingame, CA, USA) for 30 min at room temperature. Positive staining was visualized by adding 3,3′-diaminobenzidine for 1–2 min. The tissues were then counterstained with hematoxylin and dehydrated before mounting. At least two sections of each specimen were examined under light microscopy.

### Genotyping PCR

Genomic DNA was purified from the tail tip using the Wizard Genomic DNA Purification Kit (Promega, Tokyo, Japan). Genotyping PCR was performed using Tks Gflex DNA Polymerase (Takara Bio Inc., Shiga, Japan) and the primers listed in Supplementary Table S6 online. Putative off-target sites for each sgRNA were identified by CRISPOR (http://crispor.tefor.net/).

### Sperm kinetics analysis

CASA was performed using a Hamilton Thorn IVOS (ver. 12.01) CASA analyzer (Hamilton Thorn Research, Beverley, MA, USA). Spermatozoa were collected from the cauda epididymis of adult B6N or CRISPR founder mice (12–15 weeks) and preincubated in 200 μL HTF medium for 1 h in a humidified atmosphere at 37 °C in 5% CO_2_. After 50 min of sperm preincubation, the CASA machine was set up and equilibrated to 37 °C. After 1 h of preincubation, the spermatozoa suspension was loaded into a prewarmed rectangular boro tubing (0.10 mm × 2.30 mm) and inserted in the CASA machine to record quantitative parameters of sperm movement.

### Sperm staining

Spermatozoa were stained with Hoechst 33342 (for DNA) and Rhodamine 123 (for mitochondria) to evaluate the morphology of the sperm head and midpiece. The sperm mass was collected from the cauda epididymis and incubated in 200 μL HTF medium for sperm swim-up. A 10 μL aliquot of sperm suspension was collected at 5–10 min and stained with 5 μg/mL of Hoechst 33,342 and 5 μg/mL of Rhodamine 123 for 15 min at 37 °C. After washing twice with PBS, the stained sperm suspension was placed on a glass slide and covered with a cover glass. The samples were observed using Nikon C2 confocal microscopy (Nikon, Tokyo, Japan).

The plasma membrane integrity and acrosome status of spermatozoa were evaluated as described previously using PI (2.4 mM, LIVE/DEAD Sperm Viability Kit; Molecular Probes, Eugene, OR, USA) and PNA conjugated with Alexa Fluor 488 (1 mg/mL, Invitrogen, Paisley, UK)^35^. The sperm mass was collected from the cauda epididymis and incubated in 200 μL HTF medium for sperm swim-up. A 20 μL aliquot of sperm suspension was collected at 5–10 min, 1 h, and 2 h of incubation and stained with PNA and PI for 15 min in a dark box. After incubation with dyes, 5 μL of suspension was put on a glass slide and covered with a cover glass. The samples were then observed with fluorescent microscopy (BX53, Olympus, Tokyo, Japan).

## Supplementary Information

Supplementary Video 1.

Supplementary Video 2.

Supplementary Information 1.

Supplementary Information 2.

## Data Availability

The datasets generated during and/or analysed during the current study are available from the corresponding author on reasonable request.
