# Author Correction: CRISPR/Cas9-based genetic screen of SCNT-reprogramming resistant genes identifies critical genes for male germ cell development in mice

**DOI:** 10.1038/s41598-025-00865-y

**Published:** 2025-06-23

**Authors:** Most Sumona Akter, Masashi Hada, Daiki Shikata, Gen Watanabe, Atsuo Ogura, Shogo Matoba

**Affiliations:** 1 Bioresource Engineering Division, Bioresource Research Center, RIKEN, 3‑1‑1 Koyadai, Tsukuba, Ibaraki 305‑0074 Japan; 2 Cooperative Division of Veterinary Sciences, Tokyo University of Agriculture and Technology, Fuchu, Tokyo 183‑8509 Japan; 3 Research Fellow of Japan Society for the Promotion of Science, Tokyo, Japan; 4 Graduate School of Life and Environmental Sciences, University of Tsukuba, Tsukuba, Ibaraki 305‑8572 Japan; 5 The Center for Disease Biology and Integrative Medicine, Faculty of Medicine, University of Tokyo, Tokyo, 113‑0033 Japan; 6 RIKEN Cluster for Pioneering Research, Wako, Saitama 351‑0198 Japan; 7 Present Address: Laboratory of Pathology and Development, Institute for Quantitative Biosciences, The University of Tokyo, Tokyo, 113‑0032 Japan

Correction to: *Scientific Reports* 10.1038/s41598-021-94851-9, published online 29 July 2021

The original version of this Article contained an error in Figure 2, panel D, where the assembly for the image panel ‘sgTuba3b’ was inadvertently replaced with a duplicate version of the “Control” panel.

The incorrect Figure 2 and the accompanying legend appear below.

**Fig. 2:**
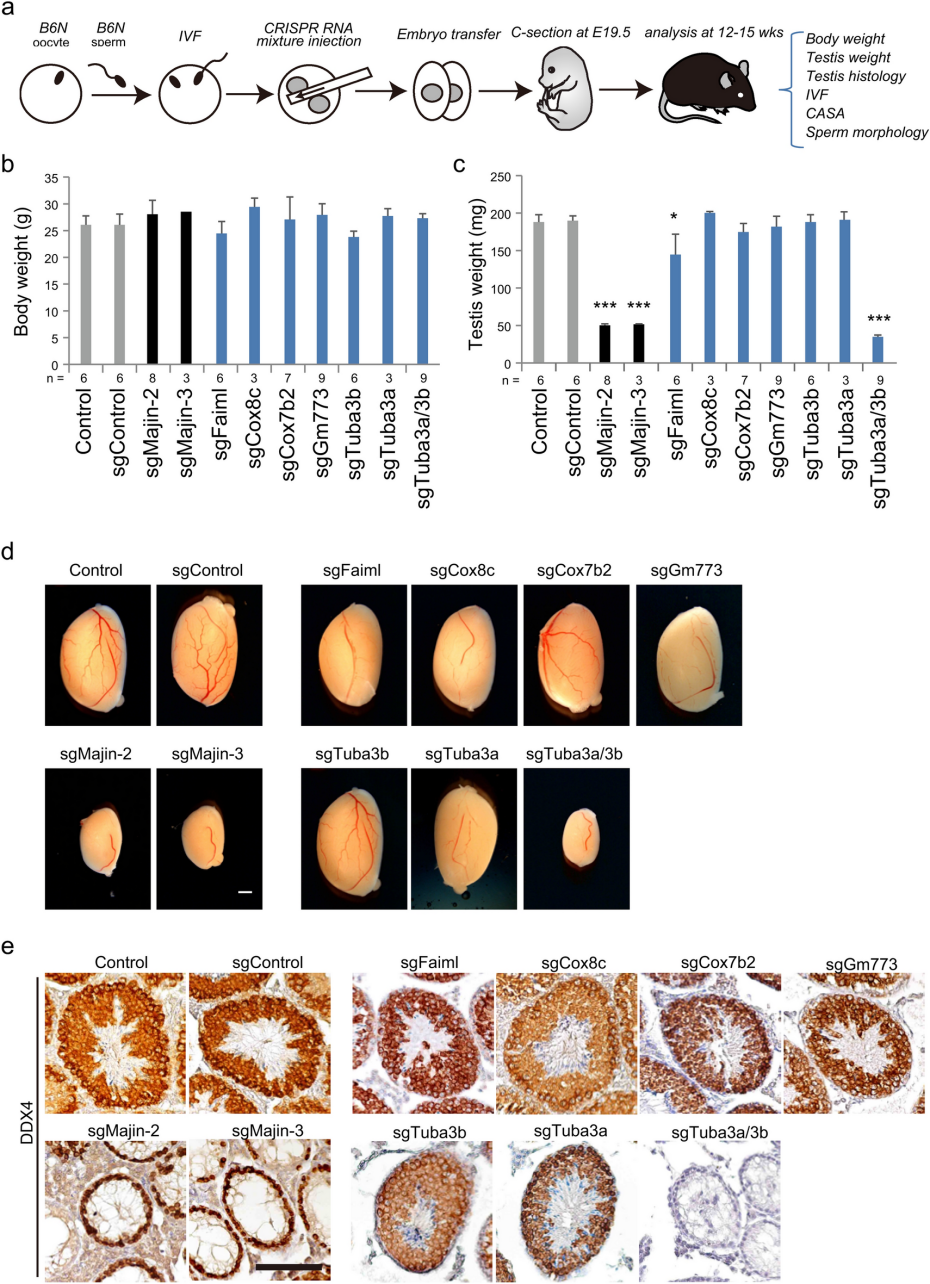
CRISPR-based genetic screening of five SRRGs. (**a**) Schematic illustration of the CRISPR-based screening procedures. C57BL/6N (B6N) oocytes and sperm were in vitro fertilized to generate B6N inbred zygotes. CRISPR mixture (*Cas9* mRNA + sgRNAs) was injected into the cytoplasm of the zygotes at 6 h post insemination (hpi). Embryos developed to the two-cell stage were transferred to the oviduct of pseudopregnant females. The embryos were recovered at E19.5 by caesarian section (C-section). The phenotypes of founder mice were screened for the indicated parameters at 12–15 weeks of age. CASA, computer-assisted sperm analysis. (**b**,**c**) Bar graphs showing the body weight (**b**) and testis weight (**c**) of adult founder mice. The testis weight was greatly reduced in sgMajin-2, sgMajin-3, and sgTuba3a/3b mice, while it was slightly reduced in sgFaiml mice. *P < 0.05, ***P < 0.001. (**d**) Gross morphology of founder testes. Note that sgMajin-2, sgMajin-3, and sgTuba3a/3b mice had significantly smaller testes than the control. Scale bar, 1 mm. (**e**) Representative immunostaining images of testis sections stained against a germ cell-specific marker, DDX4. Note that while germ cells are depleted after meiotic stages in sgMajin-2 and sgMajin-3 testis, they are completely absent in sgTuba3a/3b testis. Scale bar, 100 μm.

The original Article has been corrected.

