# Supplementary material for: CRISPR/Cas9-based genetic screen of SCNT-reprogramming resistant genes identifies critical genes for male germ cell development in mice
Source: Sci Rep. 2021 Jul 29;11:15438. doi: 10.1038/s41598-021-94851-9 (PMC8322354; doi:10.1038/s41598-021-94851-9)
Supplement: Supplementary file 3 — Supplementary Information 1. [file 41598_2021_94851_MOESM3_ESM.pdf]

SUPPLEMENTARY INFORMATION

CRISPR/Cas9-based genetic screen of SCNT-reprogramming resistant genes identifies critical genes for male germ cell development in mice

Most Sumona Akter, Masashi Hada, Daiki Shikata,
Gen Watanabe, Atsuo Ogura and Shogo Matoba

Contents:

2 Supplementary Figures
6 Supplementary Tables (Table S1 as a separate file)
2 Supplementary Videos (Video S1 and S2 as separate files)

Figure S1

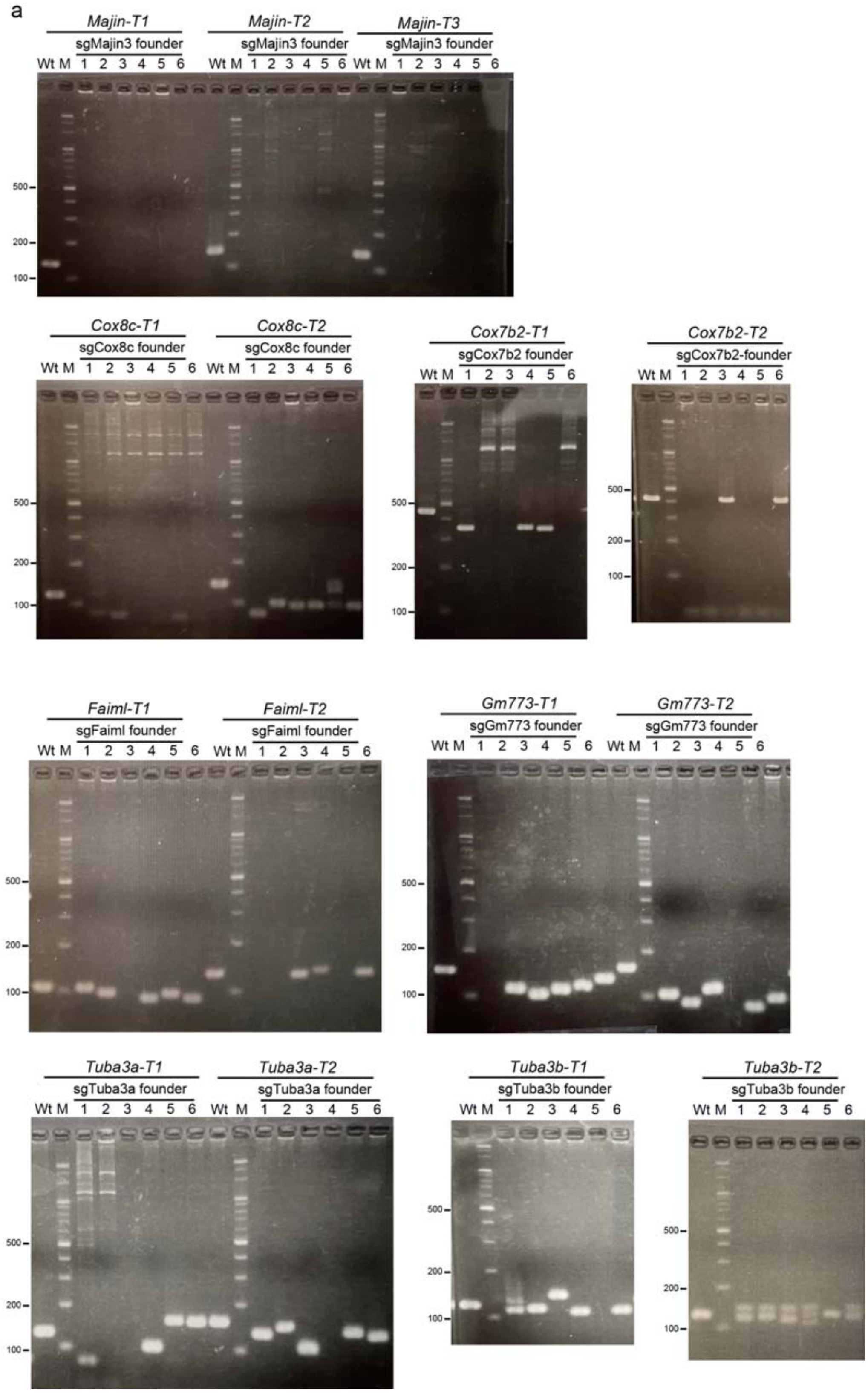
a
Majin-T1
sgMajin3 founder
Majin-T2
sgMajin3 founder
Majin-T3
sgMajin3 founder
Wt M 1 2 3 4 5 6
500
200
100
Cox8c-T1
sgCox8c founder
Cox8c-T2
sgCox8c founder
Cox7b2-T1
sgCox7b2 founder
Cox7b2-T2
sgCox7b2-founder
Faiml-T1
sgFaiml founder
Faiml-T2
sgFaiml founder
Gm773-T1
sgGm773 founder
Gm773-T2
sgGm773 founder
Tuba3a-T1
sgTuba3a founder
Tuba3a-T2
sgTuba3a founder
Tuba3b-T1
sgTuba3b founder
Tuba3b-T2
sgTuba3b founder

b

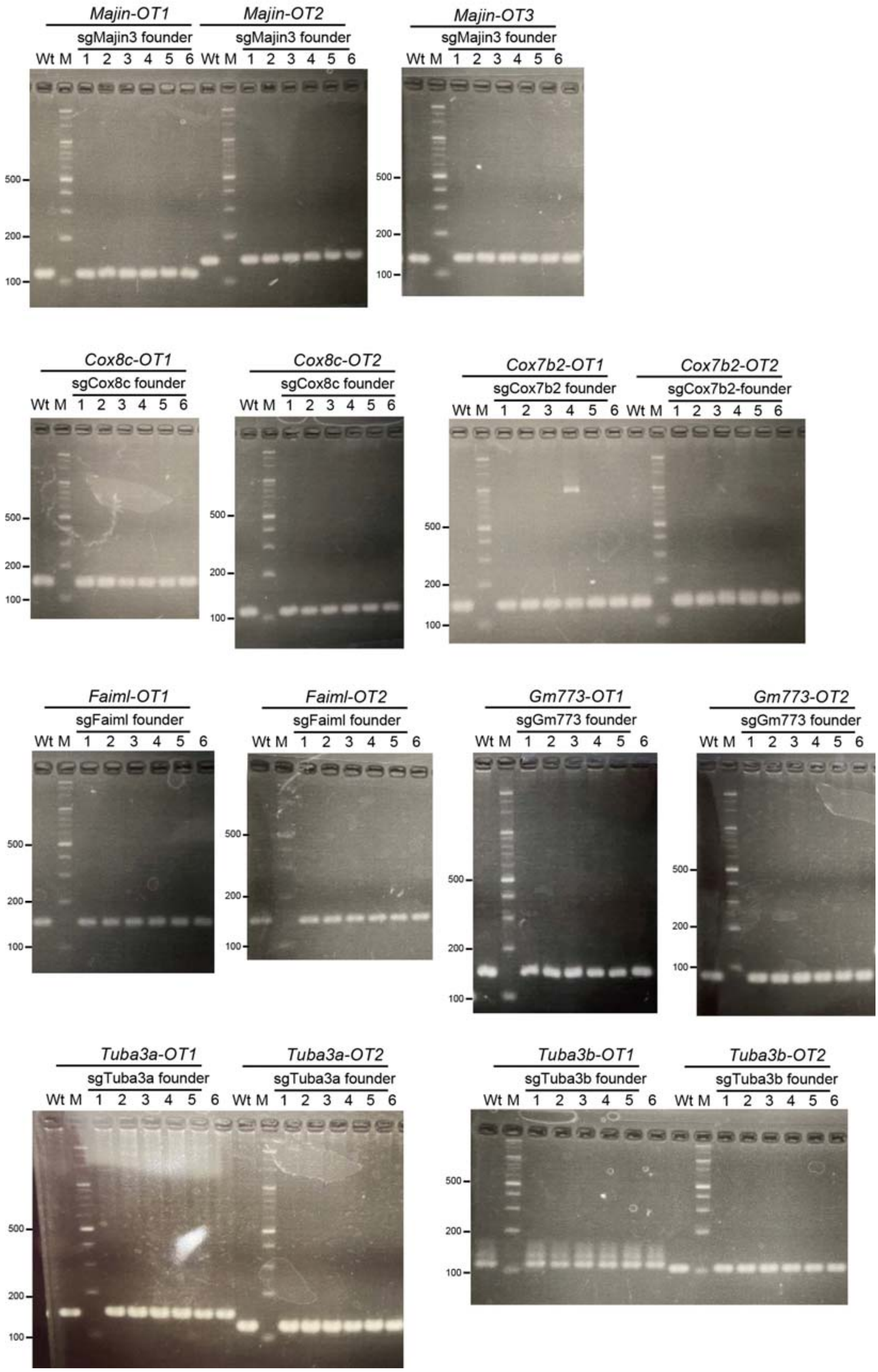
Majin-OT1
sgMajin3 founder
Majin-OT2
sgMajin3 founder
Majin-OT3
sgMajin3 founder
Wt M 1 2 3 4 5 6
500
200
100
Cox8c-OT1
sgCox8c founder
Cox8c-OT2
sgCox8c founder
Cox7b2-OT1
sgCox7b2 founder
Cox7b2-OT2
sgCox7b2-founder
Faiml-OT1
sgFaiml founder
Faiml-OT2
sgFaiml founder
Gm773-OT1
sgGm773 founder
Gm773-OT2
sgGm773 founder
Tuba3a-OT1
sgTuba3a founder
Tuba3a-OT2
sgTuba3a founder
Tuba3b-OT1
sgTuba3b founder
Tuba3b-OT2
sgTuba3b founder

Supplementary Figure S1. Highly efficient induction of indels at the target sites of the founder mice

(a) Electrophoresis images of genotyping PCR using pairs of primers that surround target sites of each sgRNA. Shown are the original gel images used in Figure 3a.

(b) Electrophoresis images of genotyping PCR using pairs of primers that surround the top ranked putative off-target site of each sgRNA. Shown are the original gel images used in Figure 3b. M, marker (100 bp ladder); Wt, wild type.

Figure S2

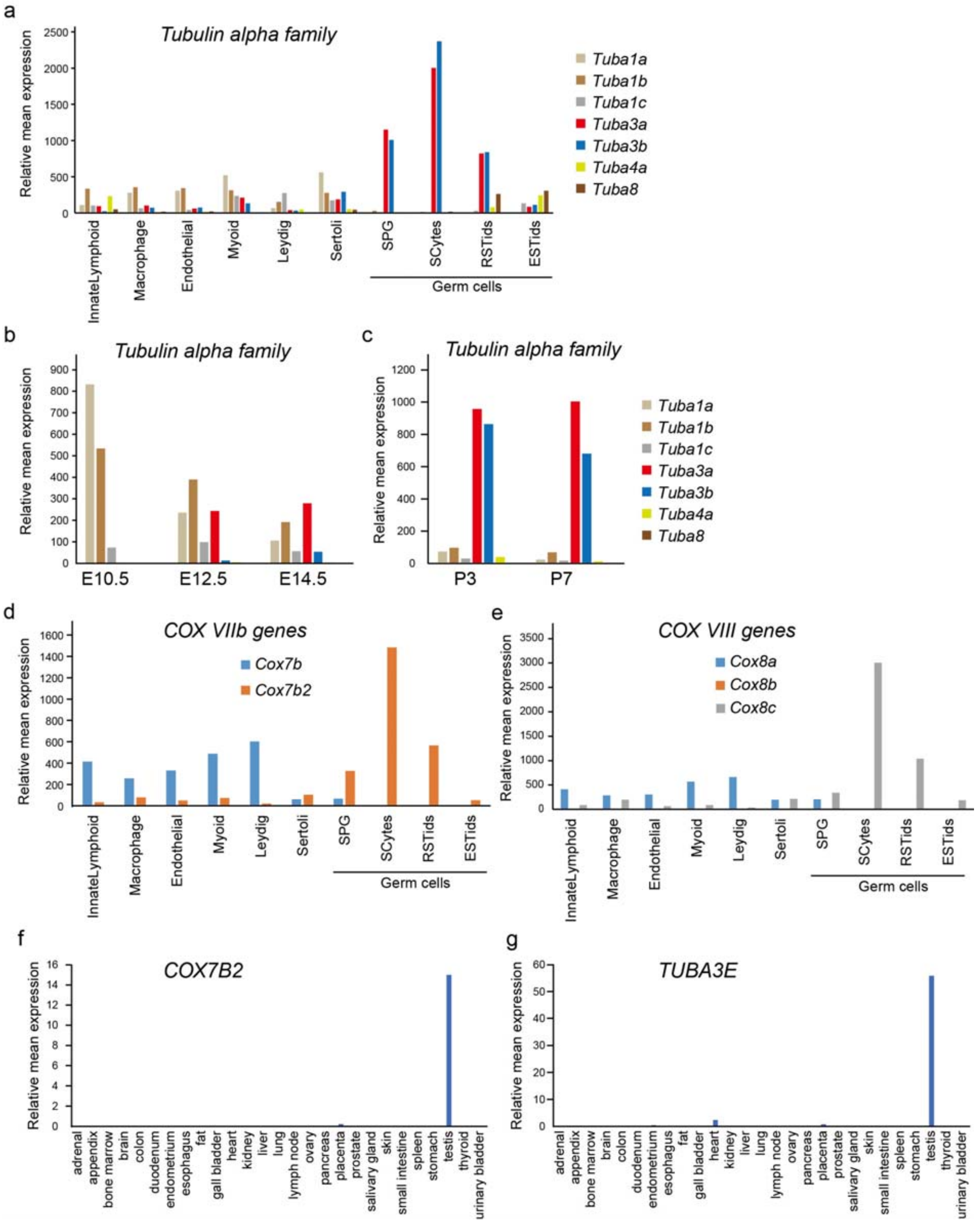
a
Tubulin alpha family
Relative mean expression
Tuba1a
Tuba1b
Tuba1c
Tuba3a
Tuba3b
Tuba4a
Tuba8
InnateLymphoid
Macrophage
Endothelial
Myoid
Leydig
Sertoli
SPG
SCytes
RSTids
ESTids
Germ cells
b
Tubulin alpha family
E10.5
E12.5
E14.5
c
Tubulin alpha family
P3
P7
d
COX VIIb genes
Cox7b
Cox7b2
e
COX VIII genes
Cox8a
Cox8b
Cox8c
f
COX7B2
g
TUBA3E
adrenal
appendix
bone marrow
brain
colon
duodenum
endometrium
esophagus
fat
gall bladder
heart
kidney
liver
lung
lymph node
ovary
pancreas
placenta
prostate
salivary gland
skin
small intestine
spleen
stomach
testis
thyroid
urinary bladder

Supplementary Figure S2. Gene expression of Tubulin alpha family genes and COX genes in the male germline

(a) Bar graphs showing the expression levels of Tubulin alpha family genes in multiple cell types of adult mouse testis. Single-cell RNA-seq data were obtained from GSE112393[2]. SPG, spermatogonia; SCytes, spermatocytes; RSTids, round spermatids; ESTids, elongated spermatids. Among eight *Tuba* genes, *Tuba3a* and *Tuba3b* were exclusively expressed in germ cells, especially in spermatogonia and spermatocytes.

(b) Bar graphs showing the expression levels of Tubulin alpha family genes in mouse embryonic male germ cells. The RNA-seq dataset was obtained from GSE76973[3].

(c) Bar graphs showing the expression levels of Tubulin alpha family genes in male mouse germ cells at P3 and P7. The RNA-seq dataset was obtained from GSE148053[4].

(d, e) Bar graphs showing the expression levels of COX VIIb (d) and COX VIII (e) family genes in multiple cell types of adult mouse testis. Single-cell RNA-seq data were obtained from GSE112393[2]. SPG, spermatogonia; SCytes, spermatocytes; RSTids, round spermatids; ESTids, elongated spermatids.

(f, g) Bar graphs showing the expression levels of *COX7B2* (f) and *TUBA3E* (g) in human tissues. RNA-seq data were obtained from PRJEB4337[5].

Supplementary Table S2. Embryonic and postnatal development of CRISPR founder mice

Group name	CRISPR target gene(s)	Injected RNA		No. of ET	Term (C-section at E19.5)			Adult (12–15 weeks old)	
		Cas9 mRNA	No. of sgRNAs		No. of implanted (% per ET)	No. of pups (% per ET)	No. of males (% per total)	No. of survived (% per born)	No. of survived males (% per total)
Control	–	–	0	105	59 (56.2)	27 (25.7)	14 (51.9)	17 (63.0)	6 (35.3)
sgControl	–	+	0	65	42 (64.6)	19 (29.2)	8 (42.1)	13 (68.4)	6 (46.2)
sgMajin-2	*Majin*	+	2	67	37 (55.2)	12 (17.9)	9 (75.0)	11 (91.7)	8 (72.7)
sgMajin-3	*Majin*	+	3	84	41 (48.8)	6 (7.1)	5 (83.3)	4 (66.7)	3 (75.0)
sgFaiml	*Faiml*	+	2	104	51 (49.0)	14 (13.5)	8 (57.1)	9 (64.3)	6 (66.7)
sgCox8c	*Cox8c*	+	2	60	34 (56.7)	14 (23.3)	4 (28.6)	11 (78.6)	3 (27.3)
sgCox7b2	*Cox7b2*	+	2	91	53 (58.2)	24 (26.4)	12 (50.0)	15 (62.5)	7 (46.7)
sgGm773	*Gm773*	+	2	181	102 (56.4)	46 (25.4)	19 (41.3)	23 (50.0)	9 (39.1)
sgTuba3b	*Tuba3b*	+	2	61	38 (62.3)	17 (27.9)	10 (58.8)	11 (64.7)	6 (54.5)
sgTuba3a	*Tuba3a*	+	2	60	33 (55.0)	14 (23.3)	5 (35.7)	11 (78.6)	3 (27.3)
sgTuba3a/3b	*Tuba3a, Tuba3b*	+	2 + 2	101	56 (55.4)	14 (13.9)	12 (85.7)	10 (71.4)	9 (90.0)

Supplementary Table S3. Development of embryos derived from CRISPR founder female mice

Female name	CRISPR targeted gene(s)	Embryo transfer at the two-cell stage*		C-section at E19.5	
		No. of transferred embryos	No. of recipients for ET	No. of implanted (% per ET)	No. of pups (% per ET)
Control	–	90	4	60 (66.7)	43 (47.8)
sgControl	–	80	4	54 (67.5)	35 (43.8)
sgMajin-2#	*Majin*	–	–	–	–
sgMajin-3#	*Majin*	–	–	–	–
sgFaiml	*Faiml*	48	3	34 (70.8)	15 (31.3)
sgCox8c	*Cox8c*	71	4	52 (73.2)	29 (40.8)
sgCox7b2	*Cox7b2*	95	5	57 (60.0)	30 (31.6)
sgGm773	*Gm773*	104	5	67 (64.4)	29 (27.9)
sgTuba3b	*Tuba3b*	83	4	49 (59.0)	24 (28.9)
sgTuba3a	*Tuba3a*	84	4	53 (63.1)	22 (26.2)
sgTuba3a/3b	*Tuba3a, Tuba3b*	115	6	57 (50.0)	17 (14.8)

*Two-cell stage embryos were generated from CRISPR founder female mice through superovulation and IVF with B6N sperm. #These mice did not produce oocytes upon superovulation.

Supplementary Table S4. RT-qPCR primers used in this study

Primer name	Sequence
Majin-RT-F1	CTCTGCAGAAACTTACGCCC
Majin-RT-R1	GTGCACATACATGGAGGCTG
Faiml-RT-F1	AATAGTAGAGTCGGTGGGCG
Faiml-RT-R1	CCTTCTCTCTTCTTCCCGCT
Cox8c-RT-F1	GCTGTTGGGATTGTCGTGTT
Cox8c-RT-R1	TTTAAAGGAGCCATGCCGTG
Cox7b2-RT-F1	ACTTCTGAGGAGAGCGTCTG
Cox7b2-RT-R1	TGCATATCTGGCCAAGGGAA
Gm773-RT-F1	TTGAGGAAATCGTGAAGCCG
Gm773-RT-R1	TCAACAGGAATAGGGGTGCC
Tuba3b-RT-F1	ATGCCAAGCGACAAAACCAT
Tuba3b-RT-R1	GTTCCGGTGCGTACTTCATC
Tuba3a-RT-F1	GCGGCTTTCAGTGGACTATG
Tuba3a-RT-R1	GGAATGCTCTAGGGTGGTGT

Supplementary Table S5. Sequence information of the primers used for the synthesis of sgRNAs

sgRNA name	Target exon	sgRNA synthesis approach *	Primer/sequence names	Sequence
Majin-sg1#	Exon 3	GeneArt	Target+PAM	GGATAAGGAAGTCATCGTCC+AGG
			Majin-sg1-F	TAATACGACTCACTATAGggataaggaagtcatcgtcc
			Majin-sg1-R	TTCTAGCTCTAAAACggacgatgacttccttatcc
Majin-sg2#	Exon 5	GeneArt	Target+PAM	AAACAAACGGGTAGGGCGTC+AGG
			Majin-sg2-F	TAATACGACTCACTATAGaaacaaacgggtagggcgtc
			Majin-sg2-R	TTCTAGCTCTAAAACgacgccctacccgtttgttt
Majin-sg3	Exon 10	GeneArt	Target+PAM	CGTTGGGGCATGGCCTGCAA+GGG
			Majin-sg3-F	TAATACGACTCACTATAGcgttggggcatggcctgcaa
			Majin-sg3-R	TTCTAGCTCTAAAACttgcaggccatgccccaacg
Faiml-sg1	Exon 2	Cloning-IVT	Target+PAM	AAACGAGTTGTGTACGTGGA+TGG
			Faiml-sg1-F	CACCGaaacgagttgtgtacgtgga
			Faiml-sg1-R	AAACtccacgtacacaactcgtttC
			Faiml-sg1-IVT	TTAATACGACTCACTATAGaaacgagttgtgtacgtgga
Faiml-sg2	Exon 3	Cloning-IVT	Target+PAM	GTCATCCAAGTGCAATACCC+AGG
			Faiml-sg2-F	CACCGgtcatccaagtgcaataccc
			Faiml-sg2-R	AAACgggtattgcacttggatgac
			Faiml-sg2-IVT	TTAATACGACTCACTATAgtcatccaagtgcaataccc
Cox8c-sg1	Exon 1	Cloning-IVT	Target+PAM	TCGAGAACAGGACTGCACGG+TGG
			Cox8c-sg1-F	CACCGtcgagaacaggactgcacgg
			Cox8c-sg1-R	AAACccgtgcagtcctgttctcgaC
			Cox8c-sg1-IVT	TTAATACGACTCACTATAGtcgagaacaggactgcacgg
Cox8c-sg2	Exon 1	Cloning-IVT	Target+PAM	AGCAGCAGGCGAGACATCGC+AGG
			Cox8c-sg2-F	CACCGagcagcaggcgagacatcgc
			Cox8c-sg2-R	AAACgcgatgtctcgcctgctgctC
			Cox8c-sg2-IVT	TTAATACGACTCACTATAGagcagcaggcgagacatcgc
Cox7b2-sg1	Exon 3	Cloning-IVT	Target+PAM	TGGGGGGTAACTCTGCCAAT+AGG
			Cox7b2-sg1-F	CACCGtggggggtaactctgccaat
			Cox7b2-sg1-R	AAACattggcagagttaccccccaC
			Cox7b2-sg1-IVT	TTAATACGACTCACTATAGtggggggtaactctgccaat
Cox7b2-sg2	Exon 3	Cloning-IVT	Target+PAM	GAGATAGTTTAGTGCATATC+TGG
			Cox7b2-sg2-F	CACCgagatagtttagtgcatatc

			Cox7b2-sg2-R	AAACgatatgcactaaactatctcC
			Cox7b2-sg2-IVT	TTAATACGACTCACTATAgagatagtttagtgcatatc
Gm773-sg1	Exon 3	GeneArt	Target+PAM	GCATTGGTTCCTACAAGAAT+GGG
			Gm773-sg1-F	TAATACGACTCACTATAGgcattggttcctacaagaat
			Gm773-sg1-R	TTCTAGCTCTAAAACattcttgtaggaaccaatgc
Gm773-sg2	Exon 7	GeneArt	Target+PAM	GCTGTGGACCTGCCGTGATG TGG
			Gm773-sg2-F	TAATACGACTCACTATAGgctgtggacctgccgtgatg
			Gm773-sg2-R	TTCTAGCTCTAAAACcatcacggcaggtccacagc
Tuba3b-sg1	Exon 3	Cloning-IVT	Target+PAM	AAGTACGCACCGGAACCTAC+CGG
			Tuba3b-sg1-F	CACCGaagtacgcaccggaacctac
			Tuba3b-sg1-R	AAACtgccgtactggacctggcggC
			Tuba3b-sg1-IVT	TTAATACGACTCACTATAGaagtacgcaccggaacctac
Tuba3b-sg2	Exon 4	Cloning-IVT	Target+PAM	CAGGGCGCCATCGAACCTTA+GGG
			Tuba3b-sg2-F	CACCGcagggcgccatcgaacctta
			Tuba3b-sg2-R	AAACtaaggttcgatggcgccctgC
			Tuba3b-sg2-IVT	TTAATACGACTCACTATAGcagggcgccatcgaacctta
Tuba3a-sg1	Exon 3	GeneArt	Target+PAM	GTCGACAATCTCTTTGCCGA+TGG
			Tuba3a-sg1-F	TAATACGACTCACTATAGgtcgacaatctctttgccga
			Tuba3a-sg1-R	TTCTAGCTCTAAAACtcggcaaagagattgtcgac
Tuba3a-sg2	Exon 4	GeneArt	Target+PAM	CCCACTGGCAACCTACGCCC+CGG
			Tuba3a-sg2-F	TAATACGACTCACTATAGcccactggcaacctacgccc
			Tuba3a-sg2-R	TTCTAGCTCTAAAACgggcgtaggttgccagtggg

* GeneArt, GeneArt Precision gRNA synthesis kit; Cloning-IVT, Cloned into px330 and *in vitro* transcription using MEGAshortscript.
# These two sgRNAs were used to produce sgMajin-2 mice.

Supplementary Table S6. Genotyping PCR primers used in this study

Primer name	Sequence
Majin-1-GT-F1	TGGTACTTCTCTGTTTCCCTTCT
Majin-2-GT-F1	GGAGAGAGTTTCCCACCTGA
Majin-3-GT-F1	GCCCTGTGGTCTCCTCTTAG
Faiml-1-GT-F1	GCAAGCACTTCTCTGTAGCA
Faiml-2-GT-F1	TCCACAGGTACAGTCCCAAC
Cox8c-1-GT-F1	CCCTTTTCTGACCTGCGATG
Cox8c-2-GT-F1	CACACTTTACCAACGGCCTT
Cox7b2-1-GT-F1	GGCGGGTTAAAACTGGTCAG
Cox7b2-2-GT-F1	GAGACAGGTTCCACTCCACA
Gm773-1-GT-F1	ACGTAGCTGAGAATGTCACTGA
Gm773-2-GT-F1	AAGAGCGTGTCATTTGCCTG
Tuba3A-1-GT-F1	GGAATTGAACAGTCCTTGCCT
Tuba3A-2-GT-F1	ACCAACCTCAACCGTCTGAT
Tuba3B-1-GT-F1	ACAGCCACCATCTAAAGCCT
Tuba3B-2-GT-F1	GACAGCTGCTCATGGTATGC
Majin-1-GT-R1	GGTGTGATTCATCCTTGACTTCA
Majin-2-GT-R1	CCCGTCAACTCCCTCTTTCT
Majin-1-GT-R1	GTGGTGGACTGCTATGTTGC
Faiml-1-GT-R1	TGAATTTGAACATGGGACCACA
Faiml-2-GT-R1	ACACTCTGGAAATTGATGGGAA
Cox8c-1-GT-R1	TTTGTGGGCTTTCTGAGTGG
Cox8c-2-GT-R1	AGGCTTCGAGAACAGGACTG
Cox7b2-1-GT-R1	AGATGGGTGTGGAGTGGAAC
Cox7b2-2-GT-R1	TGCTCCAGGAGATCTAGGGT
Gm773-1-GT-R1	ACTGAGAAGGCATTGGTTCC
Gm773-2-GT-R1	CCCAGCATCAAGAGCAACAA
Tuba3B-1-GT-R1	TCCTTCCCAGTGATGAGCTG
Tuba3B-2-GT-R1	GGAAGTGGATGCGAGGGTAT
Tuba3A-1-GT-R1	CTGGAAAGGAAGATGCAGCC
Tuba3A-2-GT-R1	TGTGTCGTCCATTACAGCCT
Majin-1-OT-F1	GCGAGCAACCTTGTACCAAT
Majin-2-OT-F1	ACCTACCCAGCTTCAGAACT
Majin-3-OT-F1	AGTCGGGCAGGGTTTTCTTA

Faiml-1-OT-F1	AGAGGCCATGGATTTGACAG
Faiml-2-OT-F1	CGACATTAGCAAGCCAACGT
Cox8c-1-OT-F1	TAGGGAACAGGACTGAACGG
Cox8c-2-OT-F1	TCCTGTGACCCTATTGATACCA
Cox7b2-1-OT-F1	CTGGGAAGAGATGAGAGAAGGT
Cox7b2-2-OT-F1	TCACGTCTTAACCTCTGATGACT
Gm773-1-OT-F1	TCTCATCAAGAATCACAGGGG
Gm773-2-OT-F1	GGAGTGCCTTTTGTCAGCA
Tuba3A-1-OT-F1	CTGGGGAGGAAAGGGTTGAT
Tuba3A-2-OT-F1	CCGTGGTTAAAGTGTGGCTC
Tuba3B-1-OT-F1	TGCTGAGTCTAGTGGCACA
Tuba3B-2-OT-F1	CCCCAAATGACGATGCCTTC
Majin-1-OT-R1	TCCATGTTTGCCAAGACTGC
Majin-2-OT-R1	CTGTGTGCCTTTCCTCTTGG
Majin-3-OT-R1	AGCCTCTATAGTTCCCTCAGG
Faiml-1-OT-R1	TCAAGCCCTCTTATTCCTACCC
Faiml-2-OT-R1	CCAACCCTTTGCCCTAATCC
Cox8c-1-OT-R1	TGTTAGACATGCCACCACCA
Cox8c-2-OT-R1	ACAGGCAAGACATAGCTGGG
Cox7b2-1-OT-R1	GTCACATGATGTTCTCTGCCC
Cox7b2-2-OT-R1	TAGAATGCGGTTATGTCCCC
Gm773-1-OT-R1	GACTCTGACATTCATTCTCCTGA
Gm773-2-OT-R1	TGCCTGCTTAAGTGCTTTCA
Tuba3B-1-OT-R1	TAGCACTACTCCATGGCCTC
Tuba3B-2-OT-R1	GAGAGGGAGCCATCAAACCT
Tuba3A-1-OT-R1	GGTCCTGAGTTCATATCCTAGCA
Tuba3A-2-OT-R1	CCTGCACAGACACAAAACCT

Supplementary Vide S1. Control sperm after 1 h of incubation in the HTF drop.
Control spermatozoa actively swam to distribute evenly within the drop.

Supplementary Vide S2. sgCox7b2 sperm after 1h of incubation in the HTF drop.
sgCox7b2 spermatozoa remained aggregated in the HTF drop even after 1 h of incubation and showed very poor motility.
